# Development and validation of the Kilifi Epilepsy Beliefs and Attitude Scale

**DOI:** 10.1016/j.yebeh.2012.06.001

**Published:** 2012-08

**Authors:** Caroline K. Mbuba, Amina Abubakar, Sally Hartley, Peter Odermatt, Charles R. Newton, Julie A. Carter

**Affiliations:** a KEMRI/Wellcome Trust Research Programme, Centre for Geographic Medicine Research (Coast), Kilifi, Kenya; b Tilburg University, The Netherlands; c Utrecht University, The Netherlands; d University of East Anglia, Norwich, UK; e Department of Epidemiology and Public Health, Swiss Tropical and Public Health Institute, Basel, Switzerland; f University of Basel, Basel, Switzerland; g Neurosciences Unit, Institute of Child Health, University College London, London, UK; h London School of Hygiene and Tropical Medicine, London, UK; i Department of Psychiatry, University of Oxford, Oxford, UK; j Centre for International Health and Development, Institute of Child Health, London, UK

**Keywords:** Epilepsy, Attitudes, Beliefs, Stigma, Africa

## Abstract

Epilepsy remains misunderstood, particularly in resource poor countries (RPC). We developed and validated a tool to assess beliefs and attitudes about epilepsy among people with epilepsy (PWE) in Kilifi, Kenya. The 50-item scale was developed through a literature review and qualitative study findings, and its reliability and validity were assessed with 673 PWE. A final scale of 34 items had Cronbach's alpha scores for the five subscales: causes of epilepsy (α = 0.71); biomedical treatment of epilepsy (α = 0.70); cultural treatment of epilepsy (α = 0.75); risk and safety concerns about epilepsy (α = 0.56); and negative attitudes about epilepsy (α = 0.76) and entire scale (α = 0.70). Test–retest reliability was acceptable for all the subscales.

The Kilifi Epilepsy Beliefs and Attitude Scale is a reliable and valid tool that measures beliefs and attitudes about epilepsy. It may be useful in other RPC or as a tool to assess the effectiveness of interventions to improve knowledge, beliefs, and attitudes about epilepsy.

## Highlights

► Kilifi Epilepsy Beliefs and Attitude Scale was developed for use in Africa. ► The scale consists of 34 items in 5 subscales. ► The scale has excellent psychometric properties.

## 1 Introduction

Negative beliefs and attitudes toward epilepsy are still prevalent among people with epilepsy (PWE), particularly in resource poor countries (RPC) [1,2] and the general public elsewhere [3–5]. Beliefs are derived culturally from previous experiences, education, families, friends and/or storytelling [6]. Attitudes are considered to develop from the evaluation of recurrent experiences within a socio-cultural context [6,7]. Lack of knowledge and negative attitudes about epilepsy affect the utilization of biomedical services, particularly the use of antiepileptic drugs (AEDs) [8,9]. Moreover, negative attitudes and beliefs may affect the quality of life of PWE more than seizures themselves [2,6,10,11].

There are a diverse range of beliefs and practices worldwide relating to the causes and treatment of epilepsy [12]. Various models have been used to describe epilepsy in Africa, Asia, South America, North America, and the Middle East [6,12–16]. Despite differences between cultures and settings, beliefs about the causes of epilepsy can be grouped into four themes: punishment for sin, bewitchment or possession, a contagious disease, and/or a disease of the brain [17]. Understanding the cultural context of epilepsy is often complicated by different terms for epilepsy, commonly on perceived differences in etiology. On the coast of Kenya, we found that the local community used different terms for seizures – ‘Nyuni’, ‘Nyago’, ‘Nyama ya dzula’ ‘Vitsala’ and ‘Kifafa’ – each of which was associated with different causes [8]. In addition, when medical explanations fail to help PWE to understand their condition, and when the prescribed medication proves ineffective in preventing seizures, they are more likely to believe in culture-specific meanings of the condition and its etiology [6].

Understanding cultural beliefs provides an insight into the way people cope with and respond to their experiences with epilepsy [17–19]. Without knowledge of these beliefs, misunderstandings and miscommunication can occur between PWE and health professionals [18], resulting in poor adherence. Therefore, it is important for health professionals to be familiar with the community's understanding about the causes and treatment of epilepsy, so that effective communication and treatment can be maintained [20–22].

We developed and validated a tool to assess beliefs and attitudes about epilepsy among PWE and their carers in Kilifi, on the coast of Kenya. We used the most appropriate information from the literature and from qualitative data to formulate a tool to represent the community's understanding of epilepsy. Developing an assessment tool based on this data enabled these views to become accessible to the health professionals, and validation allows it to be used by research planners and policy makers.

## 2 Methods

### 2.1 Development of the Kilifi Epilepsy Beliefs and Attitude Scale

The items for the scale were developed in four phases: (1) formative research and concept development; (2) item development and validity assessment; (3) revising the scale for the main survey; and (4) evaluating the scale.

#### 2.1.1 Phase 1: formative research and concept development

We conducted a literature review to locate instruments designed to measure beliefs and attitudes toward epilepsy. We searched PubMed and PsycInfo with keywords ([Epilepsy] AND [Attitudes OR Beliefs]) and identified references pertinent for the development of a scale in Kenya [6,9,23–27]. In addition, we conducted a qualitative study to explore attitudes and beliefs relating to PWE, particularly community perceptions and practices relating to epilepsy in Kilifi. We used an interview guide developed in a previous study in Kilifi [8].

Purposive sampling was used to select the participants to include representation from: PWE, their caregivers, community health workers, traditional healers, nurses, and clinicians (Table 1). Our sampling frame ensured that all groups included both sexes and that groups of children/parents of children with epilepsy covered the spectrum of disease severity. Focus group discussions and in-depth interviews were conducted by three trained interviewers fluent in KiGiriama, KiSwahili and English. The interviews were recorded, translated, and transcribed. The data were entered using NVivo qualitative analysis software (QSR; Melbourne, Vic, Australia; http://www.qsrinternational.com/) to enable storage, organization, and retrieval. The data were analyzed using framework analysis, as described by Ritchie and Spencer [28]. Themes were independently generated from the data by two researchers (CK and JC), and once thematic consensus was reached (Carter et al., in preparation), all the data were coded.

#### 2.1.2 Phase 2: item development and content validity assessment

##### 2.1.2.1 Item generation

Based on the literature review and findings from the qualitative study, a pool of 56 items was generated. Twenty-eight of the 56 items in the Kilifi Epilepsy Beliefs and Attitude Scale (KEBAS) were taken directly or adapted from questions used in previous studies investigating beliefs and attitudes toward epilepsy [6,9,23–27,29]. The remaining items were developed from the qualitative study findings. The items were grouped into five subscales: causes of epilepsy (n = 14); biomedical treatment of epilepsy (n = 13); cultural treatment of epilepsy (n = 12); risk and safety concerns about epilepsy (n = 5); and negative attitudes about epilepsy (n = 12).

Items were worded both positively and negatively within the same subscale to avoid acquiescence, affirmation, or agreement bias [30]. The scale was developed in English and translated into KiGiriama, which is the local dialect, and underwent a process of back-translation by experienced local translators.

##### 2.1.2.2 Scoring the questionnaire

The 56-item questionnaire used a 4-point Likert scale scored from 1 to 4 [31]. The scores were assigned as follows: 0 = not at all, 1 = believe a little, 2 = believe a lot, and 3 = totally believe. Positive questions were those in which ‘totally believe’ was the most positive belief or attitude with a score of ‘3’ (26 items). Reverse scoring was used for negative questions where ‘not at all’ was the most positive belief or attitude with a score of ‘3’ (30 items). This ensured that all items were scored in the same direction. The total score ranges for the five subscales were: causes of epilepsy: 0–42; biomedical treatment: 0–39; cultural treatment: 0–36; risk and safety concerns: 0–15; and negative attitudes: 0–36. Higher scores reflected more positive beliefs and attitudes about epilepsy.

##### 2.1.2.3 Face validation of the scale

Two clinicians and five research assistants with experience in working with PWE were asked to evaluate the relevance, clarity, and conciseness of the items included in the questionnaire. They were asked to determine whether the set of items accurately represented the concept under study by answering the following questions:

1. Do you think the questions measure beliefs and attitudes about epilepsy found in this community?
2. Are all these questions relevant? If not, specify which ones are irrelevant.

The seven respondents were of the opinion that the questions measured beliefs and attitudes found in the community. They also agreed on the item subscales but recommended minor revisions in the wording and structuring of some items. Based on this initial assessment, all 56 items were retained.

The questionnaire was then piloted with six PWE and seven caregivers of children with epilepsy (CWE). Two interviewers fluent in the local language administered it. The respondents were asked to: (a) comment on whether the items measured beliefs and attitudes about epilepsy; (b) rate the items on the 4-point rating scale; (c) provide explanation supporting their decision to give a certain rating (high or low) to an item; (d) comment on the time required to complete the scale; (e) comment on the clarity and flow of the questions; and (f) comment on the cultural adaptation and sensitivity of the items. The outcome indicated that six of the 56 items were not relevant for the purpose of the study (Table 2). The remaining 50 items were found to assess beliefs and attitudes about epilepsy and were reported to be clear to all the respondents. Maintaining the focus of the interviewees in the interview was a challenge, since the respondents said that the time required to complete the questionnaire (30–45 min) was too long, and they repeatedly referred to their unique experience of epilepsy during the interview. Therefore, we decided to include a vignette describing a child with epilepsy to the introduction of the scale. We adopted the vignette used in a study in North America [6] but modified it to reflect secondary generalized tonic-clonic seizures which are the most common type of seizures in the Kilifi population [32].

#### 2.1.3 Phase 3: revising the questionnaire for the main survey

We revised the scale to contain 50 items under the five conceptually and theoretically meaningful subscales: causes of epilepsy (n = 11); biomedical treatment (n = 13); cultural treatment (n = 9); risk and safety concerns (n = 5); and negative attitudes (n = 12). We maintained the 4-point Likert scale but deleted the response ‘believe a lot’ because participants did not differentiate it from the ‘totally believe’ option. We also added a ‘don't know’ response because several respondents had indicated they did not know how to answer an item. The new scores were assigned as follows: 0 = not at all, 1 = believe a little, 2 = totally believe and missing (.) = don't know. Positive questions were those in which ‘totally believe’ was the most positive belief or attitude with a score of ‘2’ (23 items). The reverse scoring was used for negative questions where ‘not at all’ was the most positive belief or attitude with a score of ‘2’ (27 items). Thus the ranges of the total scores for the five subscales were: causes of epilepsy: 0–22; biomedical treatment: 0–26; cultural treatment: 0–18, risk and safety concerns: 0–10; and negative attitudes: 0–24. Higher scores reflected more positive beliefs and attitudes about epilepsy.

#### 2.1.4 Phase 4: evaluating the scale

A descriptive cross-sectional survey was conducted in the Kilifi Health Demographic Surveillance System (KHDSS) to assess the reliability and validity of the scale. Six hundred and seventy-three PWE completed the scale, of whom 203 were PWE and 470 caregivers of CWE. The data on a subset of 65 PWE were then used to evaluate test–retest reliability of the scale, with the same interviewer administering the scale twice to the same respondents at an interval of 3 weeks.

### 2.2 Ethical considerations

Written informed consent was obtained from all study participants. Where the PWE was a child or an adult who could not respond, a caregiver was interviewed. Approval for the study was obtained from the Kenya Medical Research Institute/National Ethical Review Committee.

### 2.3 Data analysis

Data were double entered in MySQL and verified before being transferred to SPSS (version 15, SPSS Inc., Chicago) for analysis. Descriptive statistics were generated to evaluate the score distribution per response category. The internal consistency of the entire scale and subscales was calculated using Cronbach's alpha (α) [33]. An interclass correlation coefficient was used to evaluate the test–retest reliability. Confirmatory factor analysis was performed for each subscale using varimax rotation. Items were retained if they had an item-total correlation ≥ 0.2 and a factor loading ≥ 0.40 [27,34]. Correlation analysis was used to evaluate the relationship between subscale total scores, sex, and age.

To ensure that missing data did not have an undue effect on the scale, we excluded 12 items that had considerable missing information (more than 10% of the respondents had not answered) [35]. This reduced the number of items on the scale from 50 to 38. We estimated the probable values of the items that did not have substantial missing data using multiple imputation [36,37].

## 3 Results

### 3.1 Study participants

Six hundred and seventy‐three PWE completed the scale, of whom 51.0% were men. The majority of PWE, 393 (58.1%), were children aged 18 years and below. Among adults, 133 (47.5%) had no formal education, and only 8 (2.9%) had tertiary level of education. The largest faith group was traditional, which is composed of 297 (44.1%) participants (Table 3).

### 3.2 Psychometric properties of the final scale

#### 3.2.1 Descriptives

The majority of participants responded ‘totally believe’ to three subscales (causes of epilepsy, biomedical treatment, and risks and safety concerns) meaning that they had more informed beliefs about epilepsy (Table 4). In the other two subscales (cultural treatment and negative attitudes), many participants responded ‘not at all’ which also showed that they had positive beliefs since most items in the two scales were coded in reverse (Table 4).

#### 3.2.2 Internal consistency

The initial analysis demonstrated that four items had an item-total correlation < 0.2: one from the biomedical treatment subscale and three from the negative attitudes subscale: AEDs can cause side effects (− 0.05); PWE cannot lead a normal life (0.13); PWE should be resented (0.10); and PWE are a burden (0.17). After exclusion of these items, the final scale had 34 items, and alpha scores for the five subscales were: causes of epilepsy (α = 0.71); biomedical treatment (α = 0.70); cultural treatment (α = 0.75); risk and safety concerns (α = 0.56); and negative attitudes (α = 0.76) and entire scale (α = 0.70). The internal consistency of the subscales is outlined in Table 5.

#### 3.2.3 Test–retest reliability

Test–retest reliability coefficients estimated by calculating the intra-class inter-correlation coefficient were: causes of epilepsy, r = 0.64; biomedical treatment, r = 0.70; cultural treatment, r = 0.70; risk and safety concerns, r = 0.80; and negative attitudes, r = 0.81 and entire scale, r = 0.70.

#### 3.2.4 Factor analysis

The dimensionality of the scale was studied using factor analysis. Given that each subscale was conceptually derived in its development, we carried out factor analysis per subscale forcing a one‐factor solution. Our results indicated that each of these subscales could adequately be explained by a one‐factor solution. Items in each subscale had a high factor loading (≥ 0.40) as outlined in Table 6. The variance explained by each subscale was: causes of epilepsy 45.3% (eigenvalue = 2.27); biomedical treatment 36.1% (eigenvalue = 2.89); cultural treatment 33.4% (eigenvalue = 3.01); risk and safety concerns 50.2% (eigenvalue = 2.01); and negative attitudes 38.7% (eigenvalue = 3.10).

#### 3.2.5 Construct validity

Given that we had two different samples, we split the data based on who responded to the questionnaire (203 PWE and 470 caregivers). There was no difference in internal consistency based on who responded: (α = 0.79) for PWE and (α = 0.76) for caregivers.

There was no relationship between sex and any subscale scores: causes of epilepsy (*Χ*^2^ = 0.052, p = 0.819); biomedical treatment (*Χ*^2^ = 0.037, p = 0.847); traditional treatment (*Χ*^2^ = 0.145, p = 0.703); risk and safety concerns (*Χ*^2^ = 3.431, p = 0.064); and negative attitudes (*Χ*^2^ = 3.389, p = 0.066), nor between age and any subscale scores: causes of epilepsy (r = 0.02, p = 0.64); biomedical treatment (r = 0.02, p < 0.68); cultural treatment (r = 0.04, p < 0.31); risk and safety concerns (r = 0.02, p < 0.66); and negative attitudes (r = 0.14, p < 0.54).

#### 3.2.6 Beliefs and attitude scores

**Fig. 1:**
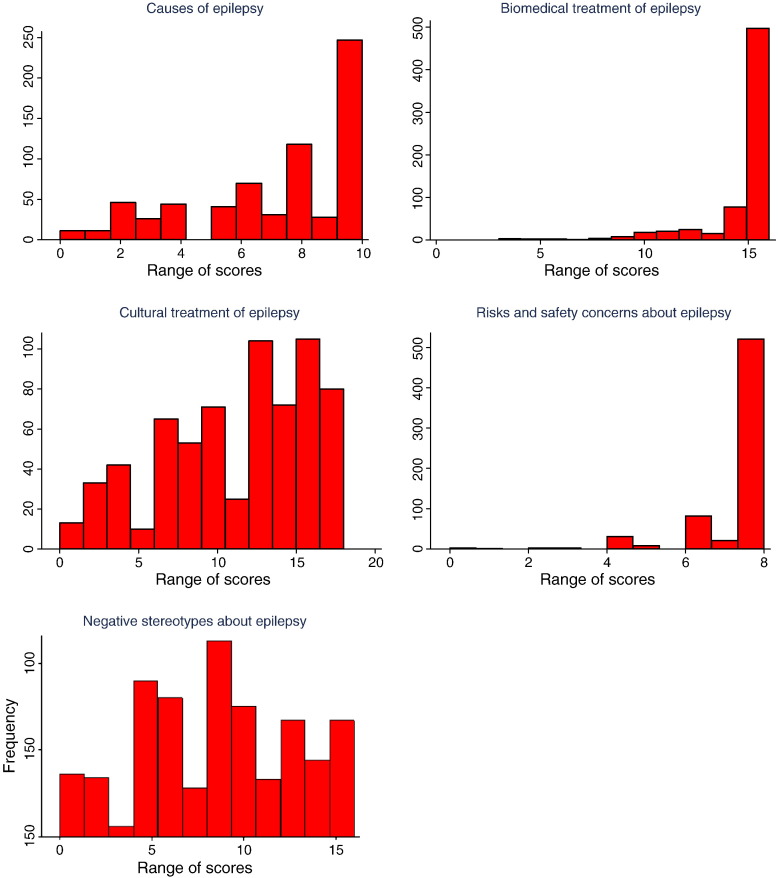
Scores for the five subscales of the Kilifi Epilepsy Beliefs and Attitude Scale.

The final scale had 34 items, and the number of items in each subscale was: causes of epilepsy (n = 5); biomedical treatment of epilepsy (n = 8); cultural treatment of epilepsy (n = 9); risks and safety concerns about epilepsy (n = 4); and negative attitudes about epilepsy (n = 8). The total score ranges for the five subscales were: causes of epilepsy 0–10; biomedical treatment 0–16; traditional treatment 0–18; risk and safety concerns 0–8; and negative attitudes 0–16 (Fig. 1). Out of the 673 respondents, the majority had positive beliefs and attitudes about epilepsy as depicted in Fig. 1: causes of epilepsy (63%); biomedical treatment (91%); cultural treatment (73%); risks and safety concerns (93%); and negative attitudes (69%).

## 4 Discussion

The purpose of this study was to develop, validate, and apply a tool to measure epilepsy beliefs and attitudes among PWE in Kilifi. Literature reviews and formative research were undertaken to identify beliefs and attitudes about epilepsy, and this led to the development of a tool that had five subscales, which represented medical and non-medical beliefs about epilepsy.

### 4.1 Reliability

Reliability analysis demonstrated an acceptable alpha score for the scale overall (α = 0.70) and the four subscales (ranging from 0.70 to 0.76), which demonstrated adequate internal consistency meeting the standard criteria for scale development [30,38]. However, the risks and safety concern subscale demonstrated poor internal consistency (α = 0.56) which could be attributed to the fewer items in this subscale or less knowledge about these issues.

The items in each subscale had acceptable item-to-total correlation (r = 0.24–0.59) [9,31], suggesting that all the items correlated well with the overall subscale scores. The test–retest reliability for three subscales was good (r-values 0.64 to 0.70) and for two subscales was excellent (0.80 to 0.81) [38]. This suggests the subscales are highly repeatable and thus reliable.

### 4.2 Validity

The confirmatory factor analysis suggests that the scale is not uni-dimensional, since it did not measure one construct; it had five subscales assessing different types of beliefs and attitudes about epilepsy. The first factor, causes of epilepsy, measured what is perceived to cause recurrent seizures. The factor on biomedical treatment looked at beliefs surrounding modern medicine, whereas the cultural treatment looked at beliefs that have a cultural orientation. The risk and safety concerns addressed activities that are perceived to be dangerous for PWE, due to the unpredictability of seizures.

The final subscale assessing negative attitudes captured beliefs that could lead to the ostracisation of PWE. The items in all the subscales had strong factor loading (≥ 0.40) [27], similar to that reported in other studies [9,27,39]. Internal consistency did not differ whether it was a PWE who responded or a caregiver. This suggests that beliefs and attitudes of children or PWE with neuro-cognitive impairment can be assessed through a caregiver using the same scale.

Sex and age were not correlated with any of the subscale scores, supporting the utility of the KEBAS as a tool that could capture differences in beliefs and attitudes among participants regardless of sex or if the information was given by PWE or their carer (as in children). These findings are supported by two other studies that showed that beliefs and attitudes did not vary by these two demographic variables [6,39], although not in another study [25].

### 4.3 Strengths

The use of a Likert scale provided a systematic method of gathering information about participants' beliefs and attitudes about epilepsy, shortening the interview time and providing numerical values, which were used to compare participants with high and low scores.

The KEBAS also had two methodological strengths that are important in interpreting the findings of acceptable psychometric properties. The first is the large sample size on which the measurement was performed. Insufficient sample sizes are a common methodological flaw in principal component and factor analyses. Antonak and Levneh recommended that when testing the properties of a scale, the sample size should be five times the number of items on the scale (i.e., 5 × 34 = 170) [23]. We had 673 respondents, lending confidence to the estimates we reported.

The second methodological strength is the excellent response rate. All the participants completed the questionnaire, which minimized the likelihood that non-responders may be systematically different than responders. This strengthens the generalizability of the findings and potentially increases the stability of the findings.

## 5 Limitations

Despite extensive efforts spent on developing and pre-testing the scale, the possibility that it does not represent accurately all possible beliefs and attitudes about epilepsy still exists. In particular, words may not be easily translated into some dialects. For example, “should not” and “cannot” are similar in KiGiriama, the local dialect. Even though the test–retest reliability was conducted within an acceptable period of 3 weeks, responses may have been biased because the respondents were sensitized by the first testing.

## 6 Conclusion

The 34-item KEBAS is a reliable and valid tool that captures beliefs and attitudes about epilepsy in a resource poor setting. It could be used as an evaluation tool to assess the effectiveness of interventions designed to increase knowledge, influence beliefs, and improve attitudes about epilepsy. To enhance the tool's utility, it should undergo further validation in different cultures and languages.

## Figures and Tables

**Table 1:** Participants involved in Phase 1.

Key informant group	Data collection method		
Focus group discussions (number of participants in parentheses)	Individual interviews	Participatory workshops (number of participants in parentheses)
Adults with epilepsy	1	1	1 PWE
(3)		(11)
Children with epilepsy	1 (6)	1	1 PWE (1)
Family members of children with epilepsy	1 mother (5) 1 father (3) 2 siblings (4, 3) 1 grandmother (3)	2 mothers 1 father 1 grandmother	1 parents and grandparents (14) 1 person with epilepsy (2 grandparents, 2 parents, 1 sibling^a^)
Biomedical service providers	2 CHWs (8, 10)	2 dispensary nurses 2 clinical officers (private clinic) 1 psychiatrist (government hospital) 1 pediatrician (government hospital)	1 traditional healer and CHWs (7 CHWs) 1 biomedical service provider and CHWs (6 CHWs, 1 nurse^b^)
Traditional service providers		3 traditional healers	1 traditional healer and CHWs (3)
Community intervention organizations		1 chairlady of ‘Maendeleo ya wanawake' (women's organization) 1 member of staff at an organization for the rehabilitation of people with disabilities	
Units of analysis (number of people)^c^	9 (45)	17 (17)	4 (48)

PWE = person with epilepsy; CHW = Community Health Worker.

a Although this participatory workshop was intended for PWE, 5 family members also accompanied some of the PWE and participated in the workshop.

b We invited other biomedical service providers to this workshop but all except one nurse said they were too busy to attend.

c Total units of analysis = 30; total number of people involved = 110.

**Table 2:** Items that were not considered relevant after piloting the Kilifi Epilepsy Beliefs and Attitude Scale.

	Item	Reason for irrelevance
1	I believe epilepsy can be a result of having water in the brain	Respondent said they have never heard of water in the brain. It was difficult to describe hydrocephalus in local language
2	I believe that having fever can cause epilepsy	Respondents interpreted fever as malaria and there was already an item on malaria/fever
3	I believe that when a child is born and the immediate sibling enters and cries before the newborn does, then the newborn can have epilepsy	The concept was not familiar to all respondents
4	I believe prayers can treat epilepsy	Question was answered on religious grounds and was not relevant to all respondents
5	I believe that drugs can cure epilepsy completely	Not clear whether the type of drugs referred to were ‘traditional or biomedical’
6	I believe there is no cure for epilepsy	The word ‘cure’ was ambiguous to some respondents as it meant being seizure free for life

**Table 3:** Demographic characteristics of study participants.

Variable	Children (< 18 years)	Adults
n = 393	n = 280
*Age (years)*		
1–5	92 (23.4)	n/a
6–10	111 (28.2)	n/a
11–18	190 (48.4)	n/a
19–30	n/a	155 (55.4)
> 30	n/a	125 (44.6)

*Sex: n (%)*		
Female	184 (46.8)	148 (52.9)
Male	209 (53.2)	132 (47.1)

*Religion: n (%)*		
Christian	167 (42.5)	128 (45.7)
Islam	52 (13.2)	29 (10.4)
Traditional	174 (44.3)	123 (43.9)

*Educational level n: (%)*		
None	173 (44.0)	133 (47.5)
Primary	194 (49.4)	122 (43.5)
Secondary	26 (6.6)	17 (6.1)
Tertiary	n/a	8 (2.9)

*Occupation: n (%)*		
Farmer	n/a	150 (53.6)
Trader	n/a	46 (16.4)
Casual	n/a	34 (12.1)
Other	n/a	50 (17.9)

*Marital status: n (%)*		
Single	n/a	77 (27.5)
Married	n/a	142 (50.7)
Separated	n/a	7 (2.5)
Divorced	n/a	17 (6.1)
Widowed	n/a	3 7(13.2)

**Table 4:** Proportion of responses to the five subscales by study participants (n = 673).

	Items of each subscale	Not at all (%)	Believe a little (%)	Totally believe (%)
*Causes of epilepsy*				
1	…Epilepsy is inherited	22.0	11.6	66.4
2	…Head injury	21.7	10.4	67.9
3	…Injury at birth	42.6	5.4	50.0
4	…Malaria/fever	10.2	7.0	82.8
5	…Brain damage	20.0	9.6	70.4

*Biomedical treatment*				
6	…It is possible to treat epilepsy	9.0	8.5	82.5
7	…AEDs should be taken continuously for them to work	3.4	2.8	93.8
8	…AEDs are available in health facilities	4.9	4.9	90.2
9	…*Nyuni* is better treated by a doctor	3.9	5.5	90.6
10	…PWE should be put in a safe place during a fit	0.2	1.0	98.8
11	…AEDs control seizures	2.5	5.2	92.3
12	…Missing AEDs can make PWE fit	9.4	4.0	86.6
13	…*Vitsala* is better treated by a doctor	3.6	3.6	92.8
14	…AEDs can cause side effects	26.3	6.1	67.6

*Cultural treatment*				
15	…PWE who are burnt never get healed	41.6	5.2	53.2
16	…*Nyuni* is treatable but not *Vitsala*	57.9	5.8	36.3
17	…*Vitsala* is better treated by a *Mganga*	62.1	11.0	26.9
18	…Pouring water on PWE during a fit treats epilepsy	59.9	8.2	31.9
19	…Smearing paraffin on PWE during a fit treats epilepsy	61.4	5.9	32.7
20	…Fumigation treats epilepsy	64.0	7.0	29.0
21	…It good to put a stick in the mouth of PWE during a fit	53.3	3.2	43.5
22	…Joints of PWE should be straightened during a fit	51.7	4.3	44
23	…*Nyuni* is better treated by a *Mganga*	70.1	9.4	20.5

*Risks and safety concerns*				
24	…PWE should not/cannot^a^ climb trees	8.9	3.4	87.7
25	…PWE should not/cannot^a^ drive	12.6	3.4	84.0
26	…PWE should avoid being near fires	1.2	1.9	96.9
27	…PWE should avoid being near water	1.6	1.5	96.9

*Negative attitudes*				
28	…PWE should not/cannot^a^ marry	48.6	13.1	38.3
29	…PWE should not/cannot^a^ go to school	58.7	7.0	34.3
30	…PWE should not/cannot^a^ have a job	47.1	9.8	43.1
31	…PWE should not/cannot^a^ lead a normal life	29.7	6.1	64.2
32	…PWE should be isolated	78.6	1.5	19.9
33	…PWE should be rejected	72.2	2.7	25.1
34	…PWE should be resented	89.9	1.6	8.5
35	…PWE are a burden	26.0	3.3	70.7
36	…PWE perform poorly in school	17.7	12.8	69.5
37	…PWE are dull	21.4	16.5	62.1
38	…PWE are mad	42.0	10.6	47.4

Items were preceded with the following phrase: *I believe….*

*PWE*: people with epilepsy; *AEDs*: antiepileptic drugs; *SD:* standard deviation.

*Nyuni*: fever provoked seizures or febrile convulsions.

*Vitsala*: a local term for epilepsy or non-fever-provoked seizures.

*Mganga*: traditional healer*.*

a This depends upon the local dialect — preferably “should not”.

**Table 5:** Internal consistency of the five subscales of the Kilifi Epilepsy Beliefs and Attitude Scale (n = 673) analyzed per subscale.

	Items of each subscale	Scale mean if item deleted	Scale variance if item deleted	Corrected item-total correlation	Alpha if item deleted
*Causes of epilepsy*					
1	…Epilepsy is inherited	6.50	3.85	0.34	0.68
2	…Head injury	6.44	3.49	0.55	0.58
3	…Injury at birth	6.66	3.22	0.47	0.63
4	…Malaria	6.27	4.11	0.44	0.64
5	…Brain damage	6.35	3.99	0.43	0.64

*Biomedical treatment*					
6	…It is possible to treat epilepsy	13.32	2.26	0.45	0.67
7	…AEDs should be taken continuously for them to work	13.17	2.83	0.36	0.68
8	…AEDs are available in health facilities	13.21	2.41	0.59	0.62
9	…*Nyuni* is better treated by a doctor	13.20	2.74	0.36	0.68
10	…PWE should be put in a safe place during a fit	13.10	3.25	0.27	0.71
11	…AEDs control seizures	13.18	2.63	0.55	0.64
12	…Missing AEDs can make PWE fit	13.27	2.67	0.24	0.72
13	…*Vitsala* is better treated by a doctor	13.17	2.68	0.52	0.65

*Cultural treatment*					
14	…PWE who are burnt never get healed	9.86	19.1	0.31	0.74
15	…*Nyuni* is treatable but not *Vitsala*	9.53	18.6	0.38	0.73
16	…*Vitsala* is better treated by a *Mganga*	9.39	18.7	0.42	0.72
17	…Pouring water on PWE during a fit treats epilepsy	9.47	18.3	0.43	0.72
18	…Smearing paraffin on PWE during a fit treats epilepsy	9.50	18.2	0.44	0.72
19	…Fumigation treats epilepsy	9.41	17.7	0.54	0.70
20	…It is good to put a stick in the mouth of PWE during a fit	9.70	18.3	0.41	0.72
21	…Joints of PWE should be straightened during a fit	9.69	18.2	0.41	0.72
22	…*Nyuni* is better treated by a *Mganga*	9.25	18.6	0.47	0.72

*Risks and safety concerns*					
23	…PWE should not/cannot climb trees^a^	5.66	0.79	0.39	0.46
24	…PWE should not/cannot drive^a^	5.70	0.69	0.41	0.46
25	…PWE should avoid being near fires	5.48	1.25	0.43	0.50
26	…PWE should avoid being near water	5.49	1.25	0.36	0.52

*Negative stereotype*					
27	…PWE should not/cannot marry^a^	7.30	14.6	0.43	0.74
28	…PWE should not/cannot go to school^a^	7.16	13.6	0.59	0.71
29	…PWE should not/cannot have a job^a^	7.37	13.7	0.56	0.72
30	…PWE should be isolated	6.81	15.2	0.43	0.74
31	…PWE should be rejected	6.93	15.0	0.41	0.75
32	…PWE perform poorly in school	7.93	15.2	0.46	0.74
33	…PWE are dull	7.82	14.4	0.57	0.72
34	…PWE are mad	7.45	15.6	0.27	0.77

Items were preceded with the following phrase: *I believe…..*

*PWE*: people with epilepsy; *AEDs*: antiepileptic drugs.

*Nyuni*: fever provoked seizures or febrile convulsions.

*Vitsala*: a local term for epilepsy or non-fever-provoked seizures.

*Mganga*: traditional healer.

a This depends upon the local dialect — preferably “should not”.

**Table 6:** Confirmatory factors analysis and factor loadings of the five subscales Kilifi Epilepsy Beliefs and Attitude Scale (n = 673).

	Item	Causes of epilepsy	Biomedical treatment	Cultural treatment	Risk concerns	Negative attitudes
1	…Epilepsy is inherited	0.54				
2	…Head injury	0.77				
3	…Injury at birth	0.69				
4	…Malaria	0.68				
5	…Brain damage	0.67				
6	…It is possible to treat epilepsy		0.62			
7	…AEDs should be taken continuously for them to work		0.46			
8	…AEDs are available in health facilities		0.78			
9	…*Nyuni* is better treated by a doctor		0.57			
10	…PWE should be put in a safe place during a fit		0.40			
11	…AEDs control seizures		0.75			
12	…Missing AEDs can make PWE fit		0.34			
13	…*Vitsala* is better treated by a doctor		0.73			
14	…PWE who are burnt never get healed			0.43		
15	…*Nyuni* is treatable but not *Vitsala*			0.51		
16	…*Vitsala* is better treated by *Mganga*			0.58		
17	…Pouring water on PWE during a fit treats epilepsy			0.59		
18	…Smearing paraffin on PWE during a fit treats epilepsy			0.60		
19	…Fumigation treats epilepsy			0.70		
20	…It is good to put a stick in the mouth of PWE during a fit			0.56		
21	…Joints of PWE should be straightened during a fit			0.56		
22	*.Nyuni* is better treated by *Mganga*			0.63		
23	…PWE should not/cannot climb trees^a^				0.55	
24	…PWE should not/cannot drive				0.58	
25	…PWE should avoid being near fires				0.84	
26	.PWE should avoid being near water				0.81	
27	…PWE cannot marry					0.59
28	…PWE should not/cannot go to school					0.74
29	…PWE should not/cannot have a job					0.72
30	…PWE should be isolated					0.57
31	…PWE should be rejected					0.56
32	…PWE perform poorly in school					0.62
33	…PWE are dull					0.72
34	…PWE are mad					0.40

Items were preceded with the following phrase: *I believe….*

*PWE*: people with epilepsy; *AEDs*: antiepileptic drugs.

*Nyuni*: fever provoked seizures or febrile convulsions.

*Vitsala*: a local term for epilepsy or non-fever-provoked seizures.

*Mganga*: traditional healer.

a This depends upon the local dialect — preferably “should not”.

## References

[1] Nyame P.K., Biritwum R.B. (1997). Epilepsy: knowledge, attitude and practice in literate urban population, Accra, Ghana. West Afr J Med.

[2] WHO (1997). Epilepsy: social consequences and economic aspects.

[3] Gambhir S.K., Kumar V., Singhi P.D., Goel R.C. (1995). Public awareness, understanding & attitudes toward epilepsy. Indian J Med Res.

[4] Radhakrishnan K., Pandian J.D., Santhoshkumar T. (2000). Prevalence, knowledge, attitude, and practice of epilepsy in Kerala, South India. Epilepsia.

[5] Rwiza H.T., Matuja W.B., Kilonzo G.P. (1993). Knowledge, attitude, and practice toward epilepsy among rural Tanzanian residents. Epilepsia.

[6] Gajjar M., Geva E., Humphries T., Peterson-Badali M., Otsubo H. (2000). A new scale to assess culture-specific beliefs and attitudes about epilepsy. Epilepsy Behav.

[7] Gerow J.R., Gerow Josh R. (1993). Social psychology. Essentials psychology: concepts and applications.

[8] El Sharkawy G., Newton C., Hartley S. (2006). Attitudes and practices of families and health care personnel toward children with epilepsy in Kilifi, Kenya. Epilepsy Behav.

[9] Martiniuk A.L., Speechley K.N., Secco M., Karen C.M. (2007). Development and psychometric properties of the Thinking about Epilepsy questionnaire assessing children's knowledge and attitudes about epilepsy. Epilepsy Behav.

[10] Atadzhanov M., Chomba E., Haworth A., Mbewe E., Birbeck G.L. (2006). Knowledge, attitudes, behaviors, and practices regarding epilepsy among Zambian clerics. Epilepsy Behav.

[11] Choi-Kwon S., Park K.A., Lee H.J. (2004). Familiarity with, knowledge of, and attitudes toward epilepsy in residents of Seoul, South Korea. Acta Neurol Scand.

[12] Andermann L.F. (1995). Epilepsy in developing countries. Transcult Psychiatry Res Rev.

[13] Banerjee T., Banerjee G. (1995). Determinants of help-seeking behaviour in cases of epilepsy attending a teaching hospital in India: an indigenous explanatory model. Int J Soc Psychiatry.

[14] Khan A., Huerter V., Sheikh S.M., Thiele E.A. (2004). Treatments and perceptions of epilepsy in Kashmir and the United States: a cross-cultural analysis. Epilepsy Behav.

[15] Placencia M., Farmer P.J., Jumbo L., Sander J.W., Shorvon S.D. (1995). Levels of stigmatization of patients with previously untreated epilepsy in northern Ecuador. Neuroepidemiology.

[16] Reis R. (1994). Anthropological aspects. Trop Geogr Med.

[17] Andermann L.F. (2000). Epilepsy in our world: an ethnographic view. Epilepsy Behav.

[18] Albert R. (1983). Exploring patient beliefs. Arch Intern Med.

[19] Desai P., Padma M.V., Jain S., Maheshwari M.C. (1998). Knowledge, attitudes and practice of epilepsy: experience at a comprehensive rural health services project. Seizure.

[20] Dekker P.A. (1998). Epilepsy: a manual for medical and clinical officers in Kenya.

[21] Kim Y.J., Synder B.O., Lai-Biker A.Y., Manoleas P. (1996). Culturally responsive psychiatric case management with South East Asians. The cross-cultural practice of clinical case management in mental health.

[22] Sue S. (1998). In search of cultural competence in psychotherapy and counseling. Am Psychol.

[23] Antonak R., Livneh H. (1988). The measurement of attitudes towards people with disability: methods, psychometrics and scales.

[24] George J., Mackinnon A., Kong D.C., Stewart K. (2006). Development and validation of the beliefs and behaviour questionnaire (BBQ). Patient Educ Couns.

[25] Lowe-Pearce C., Camfield C.S. (2005). Elementary school epilepsy survey (ESES): a new measure of elementary school students' knowledge and attitudes about epilepsy. Epilepsy Behav.

[26] Ndoye N.F., Sow A.D., Diop A.G. (2005). Prevalence of epilepsy its treatment gap and knowledge, attitude and practice of its population in sub-urban Senegal an ILAE/IBE/WHO study. Seizure.

[27] DiIorio C.A., Kobau R., Holden E.W. (2004). Developing a measure to assess attitudes toward epilepsy in the US population. Epilepsy Behav.

[28] Ritchie J., Spencer E., Bryman A., Burgess R.G. (1994). Qualitative data analysis for applied policy research. Analyzing qualitative data.

[29] George J., Vuong T., Bailey M.J., Kong D.C., Marriott J.L., Stewart K. (2006). Development and validation of the medication-based disease burden index. Ann Pharmacother.

[30] De Vellis R. (1991). Scale development: theory and applications.

[31] Aday L.A. (1996). Designing and conducting health surveys.

[32] Munyoki G., Edwards T., White S. (2010). Clinical and neurophysiologic features of active convulsive epilepsy in rural Kenya: a population-based study. Epilepsia.

[33] Cronbach L. (1951). Coefficient alpha and the internal structure of test. Psychometrika.

[34] Nunnally J., Bernstein I.H. (1994). Psychometric theory.

[35] Jarvie S., Espie C.A., Brodie M.J. (1993). The development of a questionnaire to assess knowledge of epilepsy: 1—general knowledge of epilepsy. Seizure.

[36] Allison D. (2000). Multiple imputation for missing data: a cautionery tale. Sociol Methods Res.

[37] Rubin D.B. (1976). Inference and missing data. Biometrika.

[38] Cicchetti D. (1994). Guidelines, criteria, and rules of thumb for evaluating normed and standardized assessment instruments in psychology. Psychol Assess.

[39] Kobau R., DiIorio C.A., Anderson L.A., Price P.H. (2006). Further validation and reliability testing of the attitudes and beliefs about living with epilepsy (ABLE) components of the CDC epilepsy program instrument on stigma. Epilepsy Behav.

